# Integrating Patient Safety Discussions with First-Year Doctor of Pharmacy Students in a Skills Lab Course

**DOI:** 10.3390/pharmacy12010023

**Published:** 2024-02-01

**Authors:** Kevin T. Fuji, Kimberly A. Galt

**Affiliations:** Department of Pharmacy Sciences, School of Pharmacy and Health Professions, Creighton University, Omaha, NE 68178, USA

**Keywords:** pharmacy education, patient safety, pharmacy skills laboratory, medication safety, medication errors, safe systems

## Abstract

The patient safety problem has been well established for over 20 years in the United States (U.S.), and there is a recognized focus on ensuring that health professions’ trainees receive explicit education in various patient safety principles and practices. While the literature provides examples of different approaches towards patient safety education for pharmacy students, there are few that focus on first-year pharmacy students. This educational observational study describes the implementation and evaluation of two 20 min patient safety learning activities integrated into a required pharmacy skills lab course. The first learning activity utilized a mock prescription and patient safety checklist that had students identify patient safety problems on the prescription, followed by a group discussion of implications for the patient. The second learning activity used images of common safety problems with a facilitated group discussion to have students identify systems-based solutions to those problems. Our study’s findings revealed that students were able to identify basic patient safety problems and safety solutions, although some additional foundational information may be needed, particularly for students who may not have pharmacy work experience. Additional research is needed to continue building a literature base on patient safety education approaches, particularly for first-year pharmacy students.

## 1. Introduction

The seminal patient safety report, *To Err is Human*, released by the Institute of Medicine (IOM) in 1999, established the often-cited statistic that 44,000 to 98,000 individuals in the United States (U.S.) die each year as a result of preventable medical errors [1]. Looking specifically at medication errors, it is estimated that 7000 to 9000 people die yearly due to medication errors, with a much larger number experiencing other impacts including short- and long-term harm and injury [2]. As medication experts, pharmacists play a central role in ensuring safe patient care as they optimize drug therapy through medication therapy management, medication reconciliation, patient counseling, evaluating and addressing drug interactions, appropriately dosing medications, and monitoring patient responses to medication treatments [3,4,5,6]. Pharmacists can also impact patient safety through the education of other health professionals about medications and avoiding medication errors [7,8]. However, a disconnect was noted by the IOM between the patient safety responsibilities of pharmacists and other health professionals in the practice setting and the formal education and training that is provided about patient safety and the science of safety [9]. 

As a 2016 study examining various patient safety and quality improvement competencies across health professions education showed no broad consensus about the “ideal” or “core” competencies needed for all health professionals, each profession’s accreditation standards provide patient safety guidance for health professions educators [10]. In the U.S., all Doctor of Pharmacy (Pharm.D.) programs must meet the accreditation standards established by the Accreditation Council for Pharmacy Education (ACPE). Patient safety is explicitly identified as a Required Element of the Didactic Doctor of Pharmacy Curriculum under Clinical Sciences and includes the following content: “Analysis of the systems- and human-associated causes of medication errors, exploration of strategies designed to reduce/eliminate them, and evaluation of available and evolving error-reporting mechanisms” [11].

There is a lack of standardization regarding how and when patient safety is incorporated into the pharmacy curricula [12,13,14]. Examples in the published literature include standalone courses (both required and elective) [15,16,17,18], simulation-based activities [19,20], practicing the use of tools such as root cause analysis [21,22], application of resources to identify and document medication-related problems on Advanced Pharmacy Practice Experiences [23], and interprofessional activities [24]. These different approaches to patient safety education have demonstrated effectiveness in areas including improving overall attitudes towards the importance of safety in practice, enhancing patient safety knowledge and skills, and increasing confidence in the ability to address patient safety problems [13,15,16]. Notably, all the examples listed cover a wide range of years in both the didactic and experiential pharmacy curriculum. Given the variation in approaches to patient safety education, there is an ongoing need to continue developing the literature base and provide examples of different methods that can be used to support patient safety training [12,13,25]. Of note, there is a limited literature (and no recent literature) describing patient safety education for students in their first year of training. 

The reason for the relative sparsity of educational efforts for first-year training is unclear. There may be a perception that students do not have enough clinical knowledge at the beginning of their pharmacy education to fully engage in patient safety learning activities [21]. Regardless of the reason, providing pharmacy students with foundational conceptual knowledge of patient safety principles early on in their training will allow them to apply this knowledge more effectively as their didactic clinical training, experiential training (including Introductory Pharmacy Practice Experiences), and even work experience progresses [13]. 

The purpose of this paper is to describe the development of two patient safety learning activities for pharmacy students in the first semester of their first didactic year and its integration into an existing required course. It is anticipated that the information presented in this paper will support the replication of similar types of patient safety learning activities, particularly earlier in students’ pharmacy education. 

## 2. Materials and Methods

This was an educational observational study conducted in the Pharm.D. program at the Creighton University School of Pharmacy and Health Professions. Two 20 min patient safety learning activities were integrated into Dispensing and Patient Care I, a required pharmacy skills lab course offered in the first semester of the first year in the Pharm.D. program. These patient safety learning activities were designed to facilitate student reflection on various causes of medication-related errors stemming from prescriptions and how patients are impacted when errors are not caught and generate systems-based strategies for ensuring that those same errors would not continue to recur. Additionally, these learning activities served as a precursor to the required Health Care Systems and Patient Safety course in the following semester to support the purposeful repetition of key curricular content, as suggested by the existing literature [13]. Patient safety content is not explicitly addressed in any other courses in the first semester of the first year. 

The Dispensing and Patient Care I skills lab course is structured as a once-weekly three-hour course. The first half of the course focuses on an introduction to hospital pharmacy practice, while the second half of the course, in which the patient safety learning activities were integrated, focuses on an introduction to community pharmacy practice. From an instructional perspective, the class is split, with half of the students attending the morning session and half the afternoon session. Students are placed in equal-sized groups (roughly 5–8 students per group depending on the overall class size) with each group simultaneously rotating through different activities, each of which is 20 min in length. Activities include taking live mock prescription transfers, transcribing voicemail prescriptions, performing dispensing checks, and simulated patient counseling. 

The patient safety learning activities were developed jointly between the study authors who both have formal training in patient safety and patient safety science and have published research in the area of patient safety education. One is a co-instructor of record for Dispensing and Patient Care I, while the other is a co-instructor of record for Health Care Systems and Patient Safety. One of the study authors (K.T.F.) facilitated all aspects of both patient safety learning activities. The activities remained constant from year-to-year with no change in content or approach. This study was reviewed by the Creighton University Institutional Review Board and deemed to be “Not Human Subjects Research”. 

### 2.1. Patient Safety Learning Activity #1

The first patient safety learning activity was built off of other activities in Dispensing and Patient Care I that focused on technical aspects surrounding prescriptions (e.g., ensuring that all needed information was transcribed during prescription order entry). The learning activity began with a five-minute mini lecture that emphasized the pharmacist’s role in ensuring safe patient care and the prescription/medication order as a key document to identify where errors or unsafe circumstances exist. The Medication Use Process (prescribing, transcribing, dispensing, administering, and monitoring) was presented as a framework for identifying errors and their potential downstream impacts on different parts of the process. Students were encouraged to view problems as part of a wider, dynamic system and, with prescribing being the first step in the Medication Use Process, to answer the following question: “What happens if an error in prescribing is not caught?”.

After the mini lecture, students were asked to view a mock prescription, which was designed to have a range of problems including information which was missing, incorrect, unclear, or not following best practices for ensuring patient safety (e.g., the use of trailing zeros). The prescription read “Amoxil 250.0 mg, Take until gone”. The prescription was correctly dated and included an illegible patient name (outside of the initials, which were distinguishable) and an incomplete address that included house number and street but not city and/or state. The acronym “NR” was circled on the prescription to note “no refills”, and the prescriber’s signature was what would be considered a standard semi-legible signature, with the prescriber’s clinic information at the top of the prescription. A figure of the mock prescription is not provided in this paper due to the poor reproducible image quality. 

The students were provided with a printed patient safety checklist and given five minutes to carry out the following: (1) select the patient safety problems that were present in the prescription; and (2) briefly describe the impact of the safety problem on the patient if the problem was not addressed prior to dispensing. The patient safety checklist was originally created for a federally funded study examining the integration of electronic prescribing into ambulatory care physician practices and adapted for use in the academic setting [26]. There was a total of 27 patient safety problems present on the checklist, with 10 of the problems being present in the mock prescription (Table 1 displays all the problems on the checklist with the ones present in the mock prescription in bold). The ten problems were selected to emphasize learning that had been introduced in other classes (e.g., the problem of trailing zeros was introduced in the Pharmacy Calculations course), to demonstrate synergy between different safety problems (e.g., illegible patient name along with omission of patient age or birthdate increases the opportunity for the wrong patient to receive the medication), and to highlight different types of implications resulting from these problems not being addressed (e.g., omission of dosage form and omission of route of administration would potentially have similar implications, so this overlap was avoided). 

For the remaining ten minutes of the learning activity, the faculty member facilitated a small group discussion talking through various patient safety problems that students had identified and the implications of each problem. Table 2 displays the answer key that was used by the faculty member during the small group discussion. The faculty member provided minimal prompts other than asking clarifying questions (e.g., if a student stated that the patient’s age and/or birthdate was omitted but did not offer any additional comments, the faculty member would prompt the student to describe what type of patient safety problem could arise from this information being omitted). If a student group had not identified all patient safety problems, the faculty member identified the missing problem(s) and asked the students to identify the implications of those problems for patients. At the end of the activity, all completed patient safety checklists were collected from students. All data were de-identified as no student names had been recorded on the checklists.

### 2.2. Patient Safety Learning Activity #2

The second patient safety learning activity focused on common patient safety problems in pharmacy practice, including illegible handwriting on prescriptions, look-alike and sound-alike medications, and similar packaging for different products. Similar to the first patient safety learning activity, it began with a five-minute mini lecture focused on providing a brief overview of a systems approach to patient safety. In particular, it emphasized a decreased focus on individual blame (unless negligence was present) and an increased focus on identifying systems-based solutions that introduced an increased risk for near misses and errors to occur [27]. 

The mini lecture was followed by a 15 min discussion in which various patient safety problems were represented by an image on a presentation slide and students were asked to generate systems-based solutions for each problem. Thus, solutions such as “punish the physician for writing illegibly” was not an appropriate systems-based solution as it focused on individual blame without examining the wider system that the physician was practicing in. Systems-based solutions for this problem could be the use of pre-printed forms for medication orders or a more resource-intensive option such as the adoption of electronic prescribing. The faculty member facilitated discussion and provided feedback to inappropriate solutions identified, but only described systems-based solutions that the student group was unable to identify. Table 3 displays an answer key which includes a text description of the different patient safety problem images and faculty-generated systems-based solutions for each problem. The discussion proceeded on a picture-by-picture basis. To assess the students’ ability to identify appropriate systems-based solutions for each problem, the faculty member made manual notes of the different solutions collectively identified by the student groups. 

### 2.3. Data Analysis

For Patient Safety Learning Activity #1, the patient safety checklists were analyzed descriptively with frequencies and percentages reported for each of the checklist items. As the goal of the activity assessment was not to “score” each individual student’s patient safety checklist but instead to broadly understand which patient safety problems were easier for first-year students to detect and which problems may have required additional focus, all data were aggregated. Individual year comparisons were conducted for each patient safety problem (e.g., 2015 vs. 2016, 2016 vs. 2017, etc.). The comparisons were analyzed using either a chi-square test or Fisher’s exact test depending on the expected frequencies. The significance level was adjusted to *p* ≤ 0.0024 to account for the 21 comparisons being analyzed. 

For Patient Safety Learning Activity #2, the manual notes recorded by the faculty member of student-identified systems-based solutions were aggregated for each year and then compared to the faculty-generated answer key provided in Table 3. 

## 3. Results

A total of 391 students completed the patient safety checklist and participated in both Patient Safety Learning Activities for the years 2015–2021. Table 4 provides a detailed breakdown of the number of students correctly identifying patient safety problems present in the mock prescription in Patient Safety Learning Activity #1 by year. 

Over 90% of students correctly identified five of the ten patient safety problems present in the mock prescription: omission of patient age or birthdate (97.4%), use of a trailing zero after a decimal point (94.6%), omission of quantity to dispense or duration of treatment (93.6%), illegible patient name (92.8%), and omission of route of administration (90.8%). This was followed by vagueness of instructions on prescription (89.0%), omission of dosage form (86.2%), omission of schedule for administration (82.9%), and omission of indication for medication (78.8%). Only 34.0% of students correctly identified omission of drug dose as a problem. The statistical analysis comparing each year of responses revealed no significant differences (*p* > 0.0024) for all comparisons. 

A minimal number of students identified patient safety problems that were not present in the mock prescription, with two exceptions: half of the students (50.4%) believed the prescription signature was illegible, and 17.4% felt the prescriber’s identity was unclear. 

For Patient Safety Learning Activity #2, the aggregated student-identified systems-based solutions for each year revealed that students were able to identify all systems-based solutions described in Table 2’s learning activity answer key. 

## 4. Discussion

Findings from this study indicate that patient safety learning activities with foundational- or introductory-level content can be integrated successfully with first-semester first-year Pharm.D. students. Despite core patient safety concepts such as a systems approach to errors being formally introduced for the first time using a mini lecture format directly prior to the small group discussion, students were able to fully engage with the content. Based on responses to the patient safety checklist, the vast majority of students were able to correctly identify basic patient safety problems that could be encountered in daily practice and explain the potential impact of those problems on the patient. Additionally, in Patient Safety Learning Activity #2, students were able to quickly grasp the basic concept of a systems approach to safety and apply it to generate solutions to common patient safety problems. Some of these positive findings could be due to students having past experience as pharmacy technicians or current experience as pharmacy interns; however, this information was not captured. Regardless, the discussion-based format of the activities allowed students who had prior or current pharmacy work experience to share it with their peers and learn from both the instructor and one another. These positive findings are similar to the other published study focusing on patient safety education in first-year pharmacy students [19]. 

As noted in the Introduction, it is unclear why there is a dearth of patient safety learning activities for first-year pharmacy students. However, this study supports a more focused integration of patient safety education earlier in a student’s pharmacy education. The existing literature base reveals that students are taught about patient safety problems (particularly medication errors) but are not consistently trained to identify strategies to resolve these problems [12]. If students develop a “safety mindset” early in their training, it will provide more opportunities for them to have patient safety concepts reinforced and strategies generated as they advance through their clinical education [13,14]. As noted above, many students observe errors in both clinical experiences and work settings, providing them with a background that increases their readiness for understanding and applying key patient safety concepts within the didactic setting [20]. 

Despite continued progress in patient safety education, there remain relatively few published examples overall that provide detailed guidance for pharmacy educators looking to incorporate specific activities into curricula [14]. The patient safety learning activities look to help address this gap and support the earlier integration of patient safety education. From the authors’ perspective, this was highly feasible to implement, and, although both authors have patient safety science expertise, a more basic level of knowledge and/or practice-based experience is sufficient for facilitating these types of learning activities. Additionally, the learning activities have been described in detail and answer keys have been provided to assist others who are interested in integrating similar activities into their curriculum. Utilizing short active learning-based activities is also beneficial for helping programs to address key patient safety content while not contributing to commonly described issues of curricular overload [12,28,29].

Despite the overall positive findings from these learning activities, there are some important points to address. On the patient safety checklist, the omission of drug dose was only detected by 34.0% of the students. This may indicate a confusion between the terms “strength” (meaning the amount of drug in a single dosage form) and “dose” (combining drug strength with the daily frequency of administration). As this activity was conducted with students in the first semester of their didactic education, earlier integration of patient safety concepts may necessitate the provision of basic definitions for each of these terms. 

While 78.8% of the students correctly selected omission of indication for medication, during the discussion many students noted that this information is not typically present (unlike other pieces of information which are legally required to be on the prescription), especially in community pharmacy settings. This is an example of a safety problem that was purposefully chosen by the study authors to stimulate additional discussion with students as it is a problem not easily solved even by using a systems framework for generating solutions and highlights a larger issue being faced in the health care sector (i.e., work towards achieving interoperability) [30]. The students demonstrated the ability to engage with this more complex safety problem, supporting the notion that many students have current and/or prior work experience which aids them in being able to grasp key patient safety concepts earlier in their pharmacy education. 

An illegible prescription signature was incorrectly noted as being present by half of the students. During discussion, this was a problem that also appeared to be influenced by students’ prior pharmacy work experience. Those with experience saw the prescription signature as difficult to read but fairly standard. This was another example of the benefit of discussions and peer sharing, with students who did not have work experience being able to learn from their more experienced peers. For most of the 17.4% of students who also incorrectly selected unclear prescriber identity, this selection was directly linked to their perceived illegibility of the prescriber’s signature. 

### Limitations

This study was conducted with students at a single institution, which may limit the generalizability of the findings. There were no knowledge-based assessments used; instead, one of the study authors recorded the strategies generated by the students during the discussions and compared it to faculty-generated strategies. The author may have been biased and recorded strategies in a more positive or detailed way than was actually shared by students. Finally, there was no longitudinal assessment of the degree to which students retained the understanding of key patient safety concepts. Although these learning activities were designed to support the longitudinal repetition and reinforcement of key concepts (with formal patient safety education provided in the following semester), future research should focus on the optimal methods and timing for supporting the long-term knowledge retention and application of patient safety concepts [13].

## 5. Conclusions

Two 20 min patient safety learning activities were integrated into a required pharmacy skills lab course offered in the first semester of the first didactic year. The evaluation of these activities found that students were able to fully engage with the learning activities and could recognize safety problems, describe the implications for patients, and identify potential systems-based solutions, despite only being provided mini lectures introducing key patient safety concepts. Consideration should be given to introducing students to foundational patient safety concepts earlier in their pharmacy education to maximize opportunities for students to reinforce and apply these concepts not only in didactic education but in experiential learning and their work settings. 

## Figures and Tables

**Table 1:** Patient safety problem checklist.

Patient Safety Problem
Illegible prescription signature
Unclear prescriber identity
**Illegible patient name**
Illegible prescription overall
Omission of patient name
**Omission of patient age or birthdate**
Omission of patient address
Omission of date prescription written
Omission of prescriber signature
**Omission of indication for medication**
Omission of prescription refill status
Omission of drug name
Omission of drug strength
**Omission of dosage form**
**Omission of quantity to dispense or duration of treatment**
**Omission of drug dose**
**Omission of route of administration**
**Omission of schedule for administration**
Use of an abbreviation for the drug name
Use of an abbreviation for the dose amount
Use of an abbreviation for the quantity
Use of an abbreviation for the route of administration
Use of an abbreviation for frequency of administration
Use of symbols on face of prescription
**Use of a trailing zero after a decimal point**
**Vagueness of instructions on prescription**
Wrong route of drug administration on prescription

Bolded items are problems that were present in the mock prescription.

**Table 2:** Implications of the patient safety problems present in the mock prescription.

Patient Safety Problem	Patient Safety Implications for the Identified Problem
Omission of patient age or birthdate	Students are taught in the pharmacy skills lab course to confirm patient identity with name, address, and birthdate. Frequently, individuals with the same name will reside in the same household (e.g., John Doe, John Doe Jr., John Doe III). Without a birthdate to confirm one’s identity, the incorrect person could receive the medication.
Use of a trailing zero after a decimal point	While this problem is less impactful on this prescription because there is no 2500 mg dosage form for amoxicillin, it could make a difference for a medication with a wide dosage range (and has the potential to lead to a supra-therapeutic dose).
Illegible patient name	If there are multiple patients with similar names, the incorrect patient could receive the prescribed medication. This is particularly a problem when combined with the omission of patient age or birthdate.
Omission of quantity to dispense or duration of treatment	Lack of quantity could lead to the dispensation of the incorrect amount of medication. Too much medication could encourage patients to continue a therapy for longer than necessary, which could increase the likelihood for adverse effects. However, too little medication could lead to patients not receiving a sufficiently therapeutic dosage of the medication, meaning that they would not get better, and their condition would not be adequately treated.
Omission of route of administration	Without a route of administration specified and the dosage form also being omitted, this could lead to a patient taking a medication in an incorrect manner (e.g., not specific to this mock prescription, but patients eating suppositories instead of inserting them rectally).
Vagueness of instructions on prescription	“Take daily” does not provide sufficient information about how often to take the medication. This could lead a patient to take what would be considered multiple doses at the same time to get through the medication therapy faster.
Omission of dosage form	Medications often have multiple dosage forms (in this case, amoxicillin can come in a tablet, capsule, or suspension). Certain dosage forms may be prescribed because of patient preferences or physical limitations (e.g., a suspension would be a better choice for a patient that has difficulty swallowing). Providing the incorrect dosage form could lead to patients forcing themselves to take the medication even with physical difficulty or could lead to intentional non-adherence.
Omission of schedule for administration	No instructions for when to take the medication can lead to problems. An example of this concerns whether a medication should be taken in the AM instead of the PM. A medication that is a stimulant may keep a patient up if it is taken in the PM. A medication that makes a patient drowsy that should be taken in the PM but is instead taken in the AM could lead to an accident. No schedule for administration also increases the potential for patients to double up on doses.
Omission of indication for medication	Pharmacists (particularly those in community/retail settings) frequently do not have access or are not provided with information about the indication for the medication. Because many medications have multiple indications (both FDA-approved and non-FDA-approved), without an indication it is difficult to assess the appropriateness of a therapy.
Omission of drug dose	While the drug strength is noted, without a schedule of administration, it is not possible for a pharmacist to know what the dose is for the patient. While antibiotics often have fairly standard doses, there may be slight variations in dose due to the type of infection being treated. This could lead to an inappropriate dose being provided if the pharmacist simply uses a standard dosing regimen without double checking with the prescriber.

**Table 3:** Patient safety problems and faculty-generated answer key of systems-based solutions.

Patient Safety Problem	Systems-Based Solutions for the Problem
An order for gentamicin was originally written as 60 mg I.M. q14 h to treat a Pseudomonas infection in a patient with severe renal impairment. When double-checking calculations, the prescriber determined that the order should actually have been written for q24 h. In order to make this correction a “2” was written over the “1” in 14 h. No other notations or markings were made.	Have a standard process in place for making corrections to a medication order. For example, clearly write the correction and date and initial changes that are made. Alternatively, re-write the entire order if a change needs to be made. Reinforce through training that pharmacists or pharmacy staff should call the prescriber to verify before filling a potentially confusing medication order. Implement electronic prescribing or computerized prescriber order entry.
A prescription for 8 units of Lantus was written unclearly, making the “u“ in units look like a zero and increasing the potential for the order to be perceived as 80 units.	Have a policy in place to write out doses in words instead of using Arabic numerals. Reinforce through training that pharmacists or pharmacy staff should call the prescriber to verify before filling a potentially confusing medication order. For medications that require weight-based dosing, always perform a quick double check to ensure that the prescribed dose falls into a reasonable range for the patient. Implement electronic prescribing or computerized prescriber order entry.
A vial of heparin and vial of heparin flush have very similar labels and are often placed next to one another on the pharmacy shelf despite having a 1000-fold difference in strength.	Physically separate the vials on the pharmacy shelf. Alternatively, place a sticker on the vial, label on the shelf, etc., to draw attention to the fact that this is a potentially high-risk medication and patient safety problem. If the problem recurs, report concerns to the manufacturer to advocate changing the appearance of the vials. Implement barcode scanning to confirm the correct medication is being selected and dispensed. In lieu of a barcode system, have a standard process in place to check NDC before dispensing.
Packages of amiloride, atenolol, folic acid, and tamoxifen look exactly the same because they are produced by the same manufacturer; however, they have different clinical uses.	Provide education to pharmacy staff about similar packaging for different medications to increase awareness and vigilance. Implement barcode scanning to confirm that the correct medication is being selected and dispensed. In lieu of a barcode system, have a standard process in place to check NDC before dispensing.
Hydroxyzine and hydralazine are look-alike and sound-alike medications, and this problem is further exacerbated when they are produced by the same manufacturer and, other than their names, have identical labels.	Provide education to pharmacy staff about look-alike and sound-alike medications to increase awareness and vigilance. Physically separate the packages on the pharmacy shelf as they would normally be in close proximity if organized alphabetically. Place a sticker on the bottles, label on the shelf, etc., to draw attention to the fact that these are look-alike sound-alike medications and potentially a patient safety problem. Work with the manufacturer to change the appearance of the packaging (e.g., different colored packaging, tallman lettering). Implement barcode scanning to confirm that the correct medication is being selected and dispensed. In lieu of a barcode system, have a standard process in place to check NDC before dispensing.

**Table 4:** Patient safety problems correctly detected by pharmacy students in the mock prescription.

Patient Safety Problem	2015, *n* (%) (*n* = 74)	2016, *n* (%) (*n* = 77)	2017, *n* (%) (*n* = 72)	2018, *n* (%) (*n* = 55)	2019, *n* (%) (*n* = 55)	2020, *n* (%) (*n* = 33)	2021, *n* (%) (*n* = 25)
Omission of patient age or birthdate	72 (97.3)	76 (98.7)	69 (95.8)	55 (100)	55 (100)	29 (87.9)	25 (100)
Use of a trailing zero after a decimal point	73 (98.6)	76 (98.7)	66 (91.7)	53 (96.4)	52 (94.5)	28 (84.8)	22 (88.0)
Omission of quantity to dispense or duration of treatment	69 (93.2)	73 (94.8)	66 (91.7)	53 (96.4)	50 (90.9)	30 (90.9)	25 (100)
Illegible patient name	70 (94.6)	75 (97.4)	66 (91.7)	52 (94.5)	48 (87.3)	29 (87.9)	23 (92.0)
Omission of route of administration	67 (90.5)	73 (94.8)	65 (90.3)	48 (87.3)	48 (87.3)	27 (81.8)	24 (96.0)
Vagueness of instructions on prescription	63 (85.1)	72 (93.5)	67 (93.1)	51 (92.7)	44 (80.0)	29 (87.9)	22 (88.0)
Omission of dosage form	61 (82.4)	73 (94.8)	61 (84.7)	51 (92.7)	41 (74.5)	27 (81.8)	23 (92.0)
Omission of schedule for administration	67 (90.5)	62 (80.5)	58 (80.6)	45 (81.8)	43 (78.2)	26 (78.8)	23 (92.0)
Omission of indication for medication	56 (75.7)	70 (90.9)	56 (77.8)	39 (70.9)	41 (74.5)	25 (75.7)	21 (84.0)
Omission of drug dose	19 (25.7)	31 (40.3)	25 (34.7)	19 (34.5)	18 (32.7)	14 (42.4)	7 (28.0)

## Data Availability

All data collected for this study are presented in this paper.
